# High quantile regression for extreme events

**DOI:** 10.1186/s40488-017-0058-3

**Published:** 2017-05-03

**Authors:** Mei Ling Huang, Christine Nguyen

**Affiliations:** Department of Mathematics & Statistics, Brock University, St. Catharines, Ontario, Canada

**Keywords:** Bivariate Pareto distribution, Conditional quantile, Extreme value distribution, Generalized Pareto distribution, Linear programming, Weighted loss function, primary: 62G32, secondary: 62J05

## Abstract

For extreme events, estimation of high conditional quantiles for heavy tailed distributions is an important problem. Quantile regression is a useful method in this field with many applications. Quantile regression uses an *L*_1_-loss function, and an optimal solution by means of linear programming. In this paper, we propose a weighted quantile regression method. Monte Carlo simulations are performed to compare the proposed method with existing methods for estimating high conditional quantiles. We also investigate two real-world examples by using the proposed weighted method. The Monte Carlo simulation and two real-world examples show the proposed method is an improvement of the existing method.

## Introduction

Extreme value events are highly unusual events that can cause severe harm to people and costly damage to the environment. Examples of such harmful events are stock market crashes, equity risks, pipeline failures, large flooding, wildfires, pollution and hurricanes. The response variable, *y*, of an extreme event is usually distributed according to a heavy-tailed distribution. It is important to estimate high conditional quantiles of a random variable *y* given a variable vector **x**=(1,*x*_1_,*x*_2_,…,*x*_*k*_)^*T*^∈*R* ^*p*^ and *p*=*k*+1.

The traditional mean linear regression is concerned with the estimation of the conditional expectation *E*(*y*|**x**) (Yu et al. 2003). The mean linear regression model assumes

$$\mu_{y|\mathbf{x}}=E\left(y|x_{1},x_{2},\ldots,x_{k}\right) =\mathbf{x}^{T} \boldsymbol{\beta }=\beta_{0}+\beta_{1}x_{1}+\beta_{2}x_{2}+\ldots +\beta_{k}x_{k}. $$

We estimate ***β***=(*β*_0_,*β*_1_,…,*β*_*k*_)^*T*^∈*R* ^*p*^ from a random sample {(*y*_*i*_,**x**_*i*_),*i*=1,…,*n*}, where **x**_*i*_ is the *p*-dimensional design vector and *y*_*i*_ is the univariate response variable from a continuous distribution. The least squares (LS) estimator $\widehat {\boldsymbol {\beta }}_{LS}$ is a solution to the following equation

1 $$ \widehat{\boldsymbol{\beta }}_{LS}=\text{arg} \underset{\boldsymbol{\beta }\in R^{p}}{\min}\sum_{i=1}^{n}\left(y_{i}-\mathbf{x}_{i}^{T}\boldsymbol{\beta}\right)^{2},  $$

that is, $\widehat {\boldsymbol {\beta }}_{LS}$ is obtained by minimizing the *L*_2_-distance.

The mean linear regression provides the mean relationship between a response variable and explanatory variables (Yu et al. 2003). However, there are limitations present in the conditional mean models. Outliers significantly affect the conditional mean models and as a result, it affects the measurement of the central location, which may be misleading. Also, when analyzing extreme value events, where the response variable *y* has a heavy-tailed distribution, the mean linear regression cannot be extended to non-central locations (Hao and Naiman 2007). Therefore, it cannot provide insightful information for extreme events. Quantile regression offers a more complete statistical model by specifying the changes in the high conditional quantiles and it will be used to estimate values of extreme events (Yu et al. 2003; Hao and Naiman 2007). We will study two real world examples in the following sections.

### 1.1 Snowfall in Buffalo (1994-2015)

Large snowstorms can be very hazardous to people’s safety, communities and their properties. They can significantly reduce visibility in an area, which makes it very dangerous for densely populated areas where major car accidents can happen on the road or accidents while flying can occur. A significant amount of snow, such as 12 inches (30 cm) or more, can cave in roofs of homes and buildings, standing trees can fall down on homes and cause the loss of electricity. There have been cases of deaths due to hypothermia, infections brought on by frostbite, car accidents caused due to slippery roads, heart attacks by overexertion while shoveling heavy snow and carbon monoxide poisoning from a power outage.

In 2006, Lake Storm “Aphid” was a lake-effect snowstorm that hit Buffalo, New York with a maximum snowfall of 24 inches (61 cm) and caused 19 fatalities. The snowstorm cost an estimated $530 million in damages. Recently, in November 2014, severe lake-effect snowstorm heavily impacted areas in and around Buffalo with snowfall ranging from 5-7 feet (1.5-2.1 m) and the maximum snowfall of the storms was 88 inches (220 cm). There were records of 14 heart attacks due to overexertion and roofs collapsing due to the sheer weight of the snow. The data set was obtained from National Weather Service Forecast Office (2017) (Full data is available at *http://www.weather.gov/buf*) and the daily snowfall was recorded in inches for 4478 days from January 1994 – January 2015. The snowfall was converted into centimeters and a threshold of 5 cm was considered since snowfall under 5 cm is very unlikely to cause extreme damage. As a result, there are *n*= 316 recorded data remaining. During January 1994 - January 2015, the top 10 largest daily snowfalls and maximum temperature in Buffalo, New York are shown in Table 1.

**Table 1:** The top 10 largest daily snowfalls with the maximum temperature in Buffalo, New York, from January 1994 to January 2015

Date	Snowfall (cm)	Maximum temperature (∘*C*)
December 10, 1995	86.11	–9.44
December 28, 2001	66.55	–2.78
November 20, 2000	63.25	1.67
December 27, 2001	55.63	–4.44
December 24, 2001	52.07	2.22
March 16, 2004	36.30	–2.78
October 13, 2006	35.61	9.39
March 12, 2014	35.10	3.89
March 8, 2008	33.30	–2.22
January 4, 1999	31.50	–6.67

**Fig. 1:**
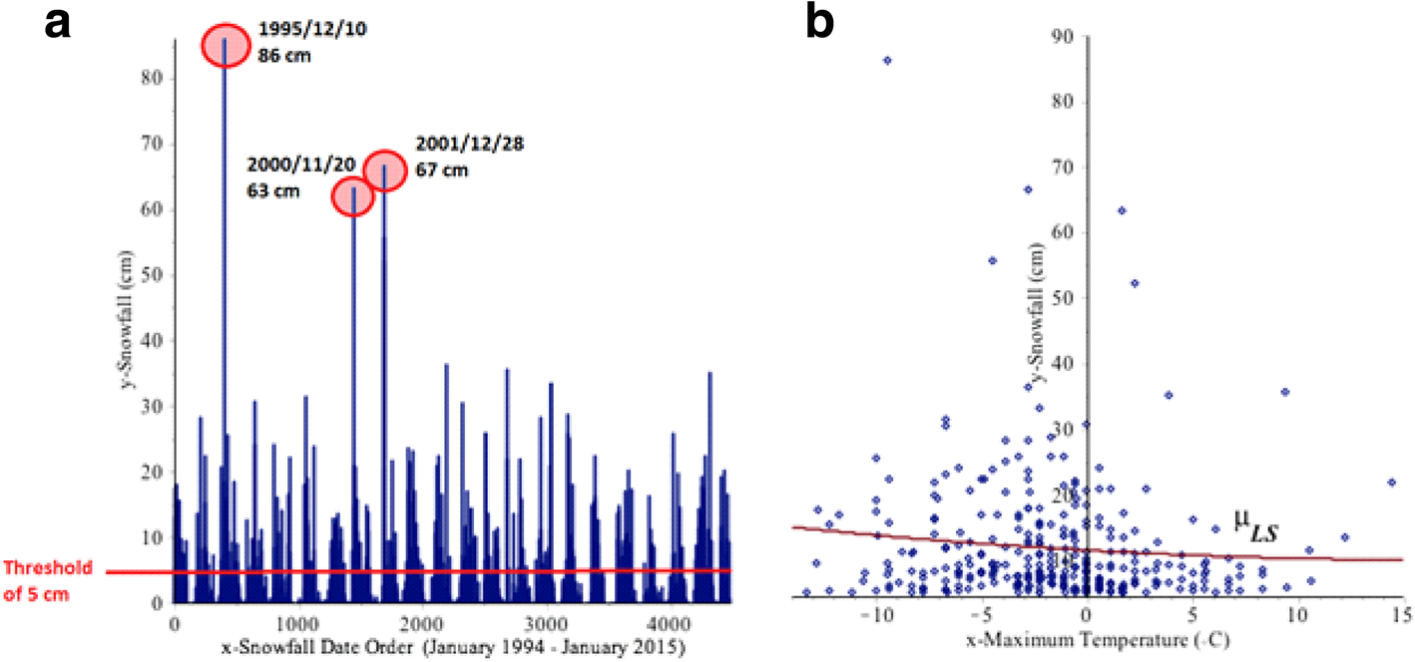
**a** The daily snowfalls (cm) in Buffalo between January 1994 and January 2015 (4478 days). **b** The scatter diagram of daily snowfalls greater than or equal to 5 cm (*n*=316 days) and least squares mean regression *μ*_*LS*_ (red) of Buffalo snowfalls given the maximum temperature between January 1994 and January 2015

In Fig. 1(a), the y-axis represents the total daily snowfall (cm) and the x-axis represents the snowfall in the order of occurrence. The maximum daily snowfall occurred on December 10, 1995 with over 86.1 cm while the average daily snowfall was 11.96 cm. Figure 1(b) shows the scatter diagram of daily snowfalls greater or equal to 5 cm. It appears that it can be modeled with a quadratic polynomial relating snowfall to maximum temperature. The least squares method was performed on a polynomial mean regression model

2 $$ E\left(y|x,x^{2}\right)=\beta_{0}+\beta_{1}x+\beta_{2}x^{2},  $$

where *y* represents the total snowfall (*cm*) and *x* represents the maximum temperature (°*C*). The red curve represents the least squares (LS) curve $\mu _{LS}= \widehat {\mu }_{y|x}=8.9879-0.2144x+0.0040x^{2}$ which was obtained by using *(1)* and the model *(2)* to estimate the mean of daily snowfall *y* for a given maximum temperature *x*. But, the least squares curve does not provide information about extreme heavy snowfalls that may cause damage. The quantile regression method will be able to estimate the high conditional quantiles. We will discuss this example further in Section 5.

### 1.2 CO_2_ Emission

Climate change is considered to be one of the most important environmental issues as it is transforming life on Earth. It affects all aspects of our natural environment including the air and water quality, health and conservation of species at risk. It has been observed that temperatures and sea levels are rising, there are stronger storms and increased damage, and increased risk of drought, fire and floods. Climate change will rapidly alter the lands and waters that we depend upon for survival and we will no longer be able to preserve our environment for our social and economic well-being.

**Fig. 2:**
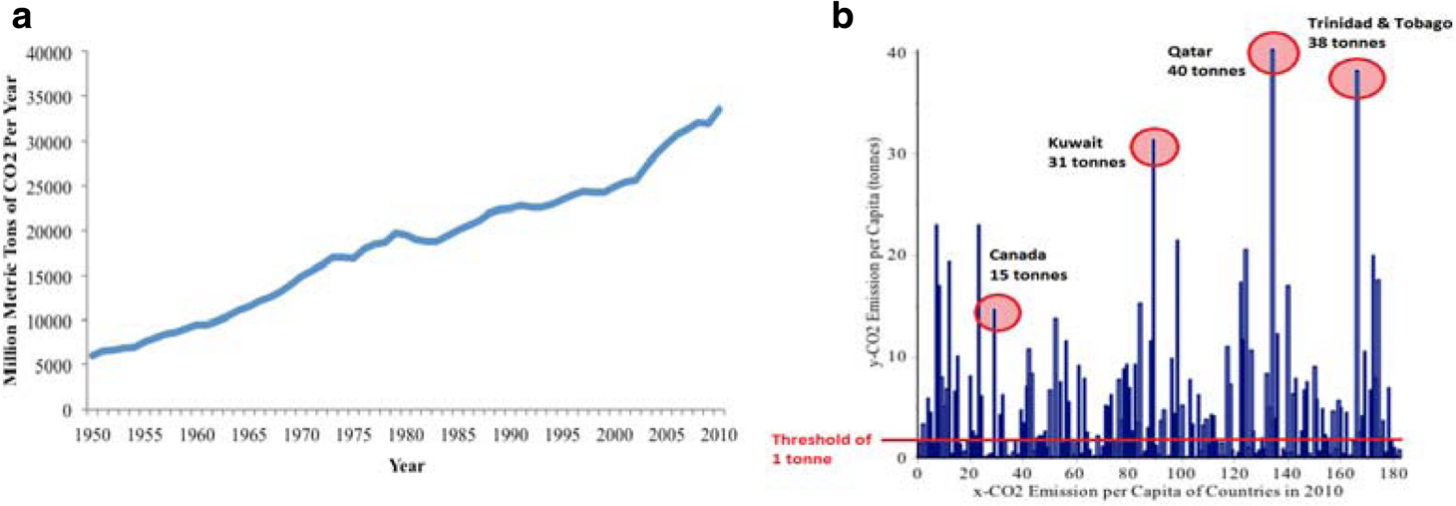
**a** Global CO_2_ Emissions (million metric tonnes) from 1950 to 2010. **b** The CO_2_ emission per capita data ordered by 181 countries in 2010

Natural processes and human activities can cause climate change. The recent global warming can be largely attributed to the carbon dioxide (CO_2_) and other greenhouse gas emissions. It was found that in 2009, CO_2_ accounted for 82% of all European greenhouse gas emissions and about 94% of the CO_2_ released to the atmosphere were from combusting fossil fuels (European Environment Agency (2017) at http://www.eea.europa.eu). Figure 2(a) shows CO_2_ emissions increases between 1950 and 2010; these increases are related with the increased energy use by an expanding economy, population and overall growth in emissions from electricity generation. It is important to estimate high conditional quantiles of the distribution of CO_2_ emission in order to prevent acceleration of climate change.

In this paper, we use the 2010 data from the Carbon Dioxide Information Analysis Center (2017) at *http://cdiac.ornl.gov* for 181 countries. The CO_2_ emission per capita was recorded in metric tonnes. There are *n*=123 countries remaining after the threshold of 1 tonne was applied. The threshold of 1 tonne of CO_2_ emission was considered since values higher than 1 tonne of CO_2_ emission would exceed the maximum allowance to emit without harming the climate. Table 2 lists the top 10 countries with the largest CO_2_ emissions and their gross domestic product (GDP) and electricity consumption (E.C.) per capita. In Fig. 2(b), the y-axis represents the CO_2_ emission per capita (tonnes) and x-axis represents the CO_2_ emission ordered by country. It can be observed in Fig. 2(b) that Qatar produced the highest CO_2_ emission per capita of 40 tonnes and Trinidad and Tobago and Kuwait produced the second and third highest CO _2_ emissions of 38 tonnes and 31 tonnes respectively. As well, several countries emitted less than 10 tonnes of CO_2_ in 2010.

**Table 2:** The top 10 countries with the largest CO_2_ emissions per capita with their GDP and Electricity Consumption (E.C.) in 2010

Country	CO_2_Emission	GDP	Electricity
	per Capita	per Capita	Consumption (E.C.)
	(tonnes)	(US $)	per Capita (kilowatts)
Qatar	40.31	71,510.16	86.01
Trinidad and Tobago	38.16	15,630.05	1657.02
Kuwait	31.32	38,584.48	913.04
Brunei	22.87	30,880.34	239.40
Aruba	22.85	24,289.14	3,262.30
Luxembourg	21.36	102,856.97	2751.26
Oman	20.41	20,922.66	1562.59
United Arab Emitrates	19.85	33,885.93	9007.35
Bahrain	19.34	20,545.97	10,142.73
United States	17.56	48,377.39	7588.42

**Fig. 3:**
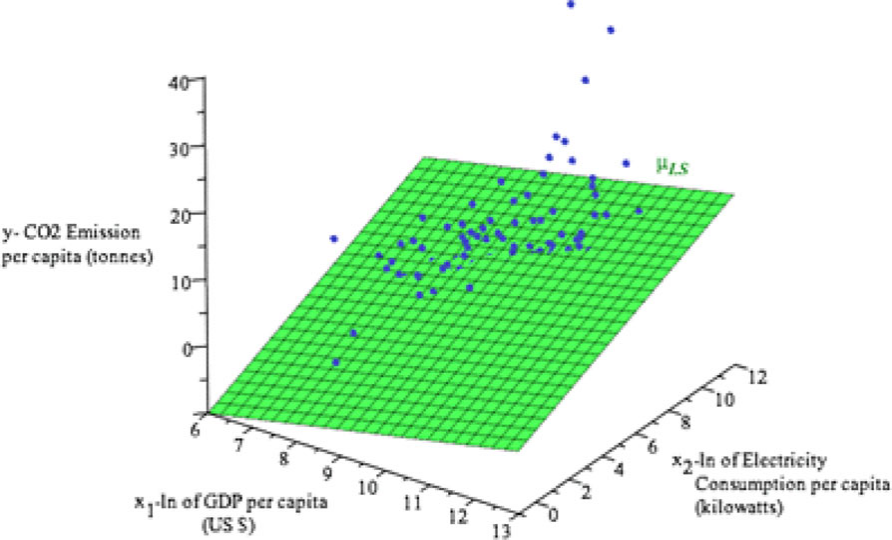
3D Scatter plot CO_2_ emission per capita given *x*_1_−ln(GDP) (US $) and *x*_2_−ln(E.C.) (kilowatts) data and LS mean regression *μ*_*LS*_ (*green*)

We set the CO_2_ emission per capita (*y*) (tonnes), ln(GDP) per capita (*x*_1_) (US $) and ln(E.C.) per capita (*x*_2_) (kilowatts) by using log-transformation. The 3D scatter diagram in Fig. 3 appears that it can be modeled using mean linear regression model *(3)* for the CO_2_ emission per capita related with the ln(GDP) and ln(E.C.). For simplification, we do not put “per capita” for these three variables in the following text.

3 $$ E\left(y|x_{1},x_{2}\right)=\beta_{0}+\beta_{1}x_{1}+\beta_{2}x_{2},  $$

**Fig. 4:**
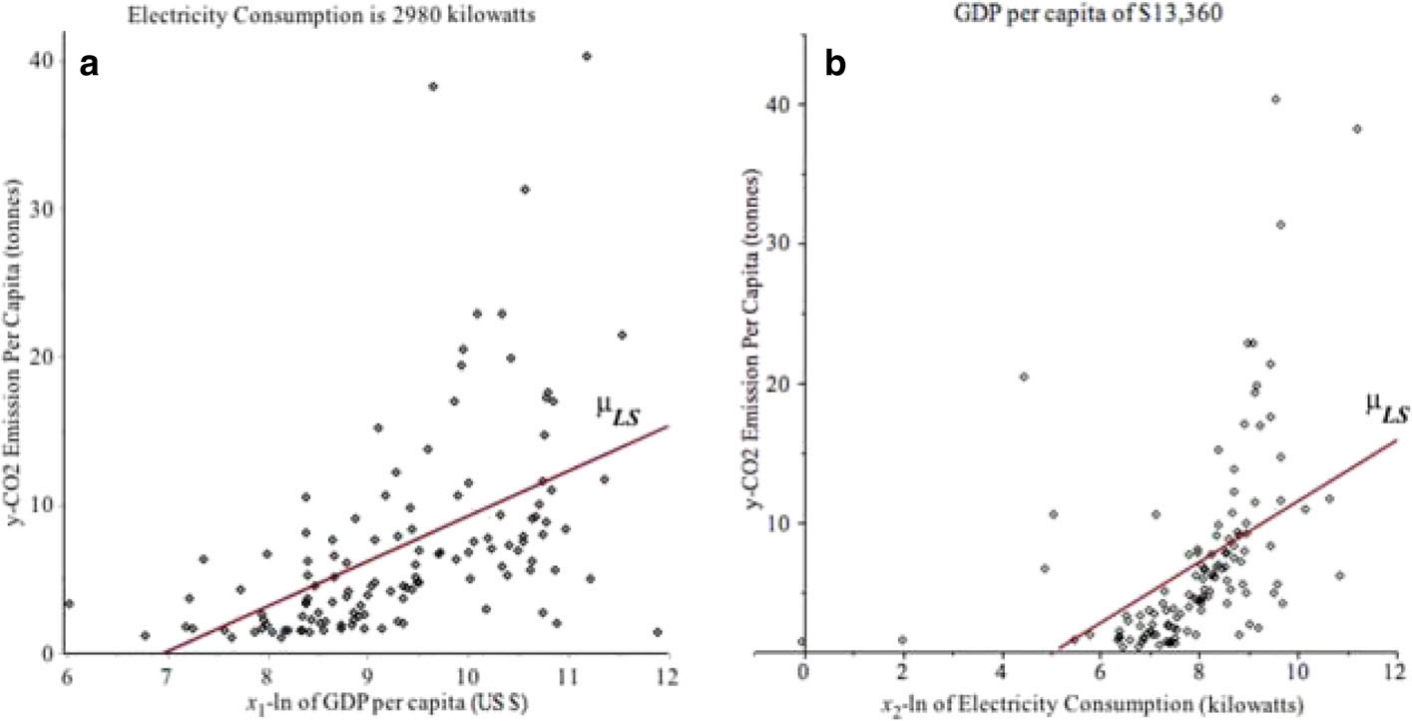
**a** Scatter plot and LS mean regression $\widehat {\mu } _{y|x_{1}}$ (red) of the CO_2_ emission per capita related to ln(GDP) *x*_1_ when the E.C. is 2980 kilowatts. **b** Scatter plot and LS mean regression $\widehat {\mu }_{y|x_{2}}$ (red) of the CO_2_ emission per capita related to ln(E.C.) *x*_2_ when GDP is $13,359.73

Figure 4(a) and (b) are 2D scatter plots. Figure 4(a) shows the relationship between ln(GDP) and CO_2_ emission per capita $\widehat {\mu }_{y|x_{1}}=-21.0984+3.0255x_{1}$ when the E.C. per capita is 2980.96 kilowatts. Figure 4(b) demonstrates the relationship between ln(E.C.) and CO _2_ emission per capita $\widehat {\mu }_{y|x_{2}}=-10.2255+2.1830x_{2}$ when the GDP per capita is $13,359.73. The least squares mean regression curves in Fig. 4 were obtained by using *(1)* and the model *(3)*.

Since the mean regression provides only the mean relationship between CO _2_ emission per capita and GDP or E.C., it cannot provide estimation for high conditional quantiles of CO_2_ emission. But the quantile regression method can estimate high CO_2_ emission quantile curves. We will discuss this example further in Section 5.

### 1.3 Main methods and results

Quantile regression is an important model with applications in many fields. At first, quantile regression provides the estimates of the conditional quantiles, which are difficult to capture by a mean regression. Second, it is also more robust against outliers in the response measurements. The objective of this paper is to study and explore a new weighted quantile regression in order to improve the existing methods. In this paper, we will do three studies:

1. The theoretical approach will be investigated.
2. Monte Carlo simulations will be performed to show the efficiency of the new weighted method relative to the existing methods.
3. The new proposed method will be applied to real-world examples on extreme events and compared to mean regression and classical quantile regression.

In Section 2, we review some notation. In Section 3, we propose an optimal weighted quantile regression method, and give its good asymptotic properties for any uniformly bounded positive weight independent of response variable *y*, with conditional density as the weight. In Section 4, the results of Monte Carlo simulations generated from the bivariate Pareto distribution show that the proposed weighted method produces high efficiencies relative to existing methods. In Section 5, the three regression methods: mean regression, classic quantile regression and proposed weighted quantile regression, are applied to the real-life examples: the Buffalo snowfall (Subsection 1.1) and CO_2_ emission (Subsection 1.2). Three goodness-of-fit tests are used to assess the distributions of the data. Studies of the examples illustrate that the proposed weighted quantile regression model fits better to the datasets than the existing quantile regression method.

## Notation

Pickands (1975) first introduced the Generalized Pareto Distribution (GPD). (Also see de Haan and Ferreira 2006).

### **Definition 2.1**

The cumulative distribution function (c.d.f.) *F*(*x*) and its corresponding probability density function (p.d.f.) *f*(*x*) of the two-parameter GPD(*γ*,*σ*) with shape parameter *γ*>0 and scale parameter *σ* of a random variable *X* are given by

4 $$ F(x)=1-\left(1+\gamma \frac{x}{\sigma }\right)^{1/\gamma },\quad \gamma,\sigma >0,\quad x>0;  $$

$$f(x)=\sigma^{-1}\left(1+\gamma \frac{x}{\sigma }\right)^{\frac{1}{\gamma } -1},\quad \gamma,\sigma >0,\quad x>0. $$

### **Definition 2.2**

The *τ*th quantile of a continuous random variable *Y* with c.d.f. *F*(*y*) is defined as

$$Q(\tau)=F^{-1}(\tau)=\inf \{y:F(y)\geq \tau \}\text{, \ }0<\tau <1. $$

### **Definition 2.3**

The *τ*th conditional linear quantile regression of *y* for given **x**=(1,*x*_1_,*x*_2_,…,*x*_*k*_)^*T*^ is defined as

$$\begin{aligned} Q_{y}\left(\tau |\mathbf{x}\right)&=Q_{\tau }\left(y|x_{1},x_{2},\ldots,x_{k}\right)=F^{-1}\left(\tau | \mathbf{x}\right)\\&=\mathbf{x}^{T}\boldsymbol{\beta }(\tau)=\beta_{0}(\tau)+\beta_{1}(\tau)x_{1}+\cdots &\!\!\!\!+\beta_{k}(\tau)x_{k},\text{\ }0<\tau <1, \end{aligned} $$

where ***β***(*τ*)=(*β*_0_(*τ*),*β*_1_(*τ*),*β*_2_(*τ*),…,*β*_*k*_(*τ*))^*T*^.

Koenker and Bassett (1978) proposed a *L*_1_−loss function to obtain estimator $\widehat {\mathbf {\beta }}(\tau)$ by solving

5 $$ \widehat{\boldsymbol{\beta}}(\tau)=\arg\min_{\boldsymbol{\beta}(\tau)\in R^{p}}\sum_{i=1}^{n}\rho_{\tau}(y_{i}-\mathbf{x}_{i}^{T}\boldsymbol{\beta } (\tau)),\text{\ }0<\tau <1,  $$

where *ρ*_*τ*_ is a loss function

$$\rho_{\tau }(u)=u(\tau -I(u<0))=\left\{ \begin{array}{r} u(\tau -1),~u<0; \\ u\tau,\ u\geq 0. \end{array} \right. $$

Quantile regression problem can be formulated as a linear program

$$\min_{(\boldsymbol{\beta }(\tau),\mathbf{u},\mathbf{v})\in R^{p}\times R_{+}^{2n}}\left\{\tau \mathbf{1}_{n}^{T}\mathbf{u}+(1-\tau)\mathbf{1}_{n}^{T} \mathbf{v}|\boldsymbol{X\beta }(\tau)+\mathbf{u}-\mathbf{v}=\mathbf{y}\right\}, $$

where $\mathbf {1}_{n}^{T}$ is an *n*-vector of 1s, **X** denotes the *n*×*p* design matrix, and **u** **,** **v** are *n* × 1 vectors with elements of *u*_*i*_,*v*_*i*_ respectively (Koenker 2005).

## Proposed weighted quantile regression

### 3.1 Proposed weighted quantile regression

Huang et al. (2015) proposed a weighted quantile regression method

6 $$ \widehat{\boldsymbol{\beta }}_{w}(\tau)=\mathbf{\arg }\min_{\boldsymbol{\beta} (\tau)\in R^{p}}\sum_{i=1}^{n}w_{i}\left(\mathbf{x}_{i},\tau \right)\rho_{\tau}\left(y_{i}-\mathbf{x}_{i}^{T}\boldsymbol{\beta }(\tau)\right),\;0<\tau <1,  $$

where *w*_*i*_(**x**_*i*_,*τ*) is any uniformly bounded positive weight function independent of *y*_*i*_, *i*=1,…,*n*, for **x**_*i*_=(1,*x*_*i*1_,*x*_*i*2_,…,*x*_*ik*_)^*T*^.

In this paper, we propose a weight as the local conditional density *f*(*y*|**x**) of *y* for given **x** at the *τ*th quantile point *ξ*_*i*_(*τ*,**x**_*i*_), which is

7 $$ w_{i}\left(\mathbf{x}_{i},\tau \right)=f_{i}\left(\xi_{i}\left(\tau,\mathbf{x}_{i}\right)\right),\;i=1,2,\ldots,n,\;0<\tau <1,  $$

where *f*_*i*_(*ξ*_*i*_(*τ*,**x**_*i*_)) is uniformly bounded at the quantile points *ξ*_*i*_(*τ*,,**x**_*i*_).

The following are the four reasons for the proposed weight in *(7)*:

1. Koenker (2005, Chapter 5, Subsection 5.3) suggested that when the conditional densities of the response are heterogeneous, it is natural to consider whether weighted quantile regression might lead to efficiency improvements.
2. The error function $\rho _{\tau }(y_{i}-\mathbf {x}_{i}^{T}\boldsymbol {\beta } (\tau))$ in *(6)*is an absolute error measure between *y*_*i*_ and the *τ*th conditional quantile *ξ*_*i*_(*τ*,**x**_*i*_) at the *i* th sample point (*y*_*i*_,**x**_*i*_), *i*=1,2,…,*n*. *f*_*i*_(*ξ*_*i*_(*τ*,**x**_*i*_)) can be interpreted as providing the local relative likelihood that the response varaibale *y* takes values in a neighborhood of *y*=*ξ*_*i*_(*τ*,**x**_*i*_):(*ξ*_*i*_(*τ*,**x**_*i*_)−*ε*, *ξ*_*i*_(*τ*,**x**_*i*_)+*ε*), for small *ε*>0. Giving the weight *f*_*i*_(*ξ*_*i*_(*τ*,**x**_*i*_)) on $\rho _{\tau }(y_{i}-\mathbf {x}_{i}^{T} \boldsymbol {\beta }(\tau))$ will make the total error $\sum _{i=1}^{n}w_{i}(\mathbf {x}_{i},\tau)\rho _{\tau }(y_{i}-\mathbf {x}_{i}^{T}\boldsymbol {\beta } (\tau))$ more reasonable.
3. The weighted estimator $\widehat {\boldsymbol {\beta }}_{w}(\tau)$ in *(6)*using weight *(7)*has good properties, which we will discuss in Subsection 3.2 below.
4. Is weight *(7)* an optimal weight? It is a difficult problem in the field as described in Chapter 5, Subsection 5.3 in Koenker (2005). Also, *f*_*i*_(*ξ*_*i*_(*τ*,**x**_*i*_)) is difficult to estimate. In this paper, we explore these two difficulties, we estimate the *f*_*i*_(*ξ*_*i*_(*τ*,**x**_*i*_)), then obtain positive results by using weight *(7)*.

In Section 4, in simulations, we compare using weight *(7)*with the weight given in Huang et al. (2014) as

8 $$ w_{i}\left(\mathbf{x}_{i},\tau \right) =\frac{\|\mathbf{x}_{i}\|^{-1}}{\sum\limits_{i=1}^{n}\|\mathbf{x}_{i}\|^{-1}},\quad 0<\tau <1,  $$

where *w*_*i*_(**x**_*i*_,*τ*)∈ [ 0,1] and $ \sum \limits _{i=1}^{n}w_{i}(\mathbf {x}_{i},\tau)=1,\;i=1,\ldots,n, \left \vert \left \vert \mathbf {x}_{i}\right \vert \right \vert =\sqrt { x_{i1}^{2}+x_{i2}^{2}+\cdot \cdot \cdot +x_{ik}^{2}},$ *k* is the number of regressors.

In this paper, we are looking for improvement of efficiency by using weights *(7)* in Section 4 simulations, and applications of the Buffalo snowfall and CO_2_ emission examples in Section 5.

### 3.2 Properties of weighted quantile regression

The following regularity conditions are necessary in deriving the asymptotic distribution of $\widehat {\boldsymbol {\beta }}_{n(w)}(\tau)$ in *(6)*with weight *w*_*i*_(**x**_*i*_,*τ*)=*f*_*i*_(*ξ*_*i*_(*τ*,**x**_*i*_)), *i*=1,2,…, 0<*τ*<1, in *(7)*. Let *Y*_1_,*Y*_2_,… be independent random variables with distribution function *F*_1_,*F*_2_,….

#### **Condition 1**

***(C1).*** The *F*_*i*_’s are absolutely continuous, with continuous densities *f*_*i*_(*ξ*) uniformly bounded away from 0 and *∞* at the quantile points *ξ*_*i*_(*τ*,**x**_*i*_),*i*=1,2,….

#### **Condition 2**

***(C2).*** There exist positive definite matrices **D**_0_(*τ*) such that the following three subconditions are satisfied

- ${\lim }_{n\rightarrow \infty }n^{-1}\sum f_{i}^{2}(\xi _{i}(\tau, \mathbf {x}_{i}))\mathbf {x}_{i}\mathbf {x}_{i}^{T}=\mathbf {D}_{0}(\tau),$ and
- $\lim \limits _{i=1,..,n}\left \Vert f_{i}(\xi _{i}(\tau,\mathbf {x} _{i}))\right \Vert $/$\sqrt {n}\rightarrow 0.$

We have the main asymptotic results for $\widehat {\mathbf {\beta }} _{n(w)}(\tau)$ in *(6)* using weight *(7).* In this case, we let $\widehat {\boldsymbol {\beta }}_{n(w)}(\tau)=\widehat {\boldsymbol {\beta }}_{n(f)}(\tau)$ in the following theorem.

#### **Theorem 3.1**

Under Conditions C1 and C2, we have

$$\sqrt{n}\left(\widehat{\boldsymbol{\beta }}_{n(f)}(\tau)-\boldsymbol{\beta }(\tau)\right) \overset{\mathcal{D}}{{\LARGE \rightarrow }} N(0,\;\tau (1-\tau)\mathbf{D}_{0}^{-1}(\tau)), \textit{ as } n\rightarrow \infty. $$

The proof of Theorem 3.1 is similar as the proof has been provided in Huang et al. (2015).

### 3.3 Comparison of quantile regression models

In order to compare the regular and weighted quantile regression models in *(5)*and *(6),* we extend the idea of measure of goodness of fit by Koenker and Machado (1999), and suggest the use of a Relative *R*(*τ*), which is defined as

9 $$ Relative~R(\tau)=1-\frac{V_{weighted}(\tau)}{V_{regular}(\tau)},\quad -1\leq R(\tau)\leq 1,  $$

where

$$V_{regular}(\tau)=\sum_{y_{i}\geq \mathbf{x}_{i}^{T}\widehat{\boldsymbol{\beta}}(\tau)}\frac{\tau}{n}\left\vert y_{i}-\mathbf{x}_{i}^{T}\widehat{\boldsymbol{\beta }}(\tau)\right\vert+\sum_{y_{i}<\mathbf{x}_{i}^{T}\widehat{\boldsymbol{\beta }}(\tau)}\frac{(1-\tau)}{n}\left\vert y_{i}-\mathbf{x}_{i}^{T}\widehat{\boldsymbol{\beta }}(\tau)\right\vert, $$

where $\widehat {\mathbf {\beta }}(\tau)$ is obtained by *(5).*

$$V_{weighted}(\tau)=\sum_{y_{i}\geq \mathbf{x}_{i}^{T}\widehat{\boldsymbol{\beta }}_{w}(\tau)}w_{i}\tau \left\vert y_{i}-\mathbf{x}_{i}^{T}\widehat{\boldsymbol{ \beta }}_{w}(\tau)\right\vert +\sum_{y_{i}<\mathbf{x}_{i}^{T}\widehat{ \boldsymbol{\beta }}_{w}(\tau)}w_{i}(1-\tau)\left\vert y_{i}-\mathbf{x}_{i}^{T} \widehat{\boldsymbol{\beta }}_{w}(\tau)\right\vert, $$

where *w*_*i*_ and $\widehat {\mathbf {\beta }}_{w}(\tau)$ are given by *(6).*

## Simulations

In this Section, Monte Carlo simulations are performed. We generate *m* random samples with size *n* each from the bivariate Pareto distribution (Mardia 1962) with c.d.f.

10 $$ F(x,y)=1-x^{-\alpha }-y^{-\alpha }-(x+y-1)^{-\alpha },\;x>1,\;y>1,\;\alpha >0,  $$

and the conditional quantile function of *(10)* is

11 $$ \xi (\tau |x)=Q_{y}(\tau |x)=1-x\left(1-\frac{1}{(1-\tau)^{-1/\left(\alpha +1\right)}}\right),x>1,\;\alpha >0,\quad 0<\tau <1.  $$

The conditional density of *y* for given *x* is

$$f(y|x)=\frac{(\alpha +1)x^{(\alpha +1)}}{(x+y-1)^{(\alpha +2)}},\;x>1,\;y>1,\;\alpha >0, $$

and the *τ*th conditional density of *y* for given *x* at the *τ*th quantile is

12 $$ f(\xi (\tau |x))=\frac{(\alpha +1)(1-\tau)^{(\alpha +2)/(\alpha +1)}}{x},\;x>1,\;\alpha >0,\quad 0<\tau <1.  $$

Assume that the true conditional quantile is *Q*_*y*_(*τ*|*x*)=*β*_0_(*τ*)+*β*_1_(*τ*)*x*. We use two quantile regression methods:

- The regular quantile regression *Q*_*R*_(*τ*|*x*) estimation based on *(5),*
  13 $$ Q_{R}(\tau |x)=\widehat{\beta }_{0}(\tau)+\widehat{\beta }_{1}(\tau)x  $$
- The weighted quantile regression *Q*_*W*_(*τ*|*x*) estimation based on *(6)*
  14 $$ Q_{W}(\tau |x)=\widehat{\beta }_{w0}(\tau)+\widehat{\beta }_{w1}(\tau)x.  $$
  For each method, we generate size *n*=300,*m*=1,000 samples. *Q*_*R*,*i*_(*τ*|*x*) or *Q*_*W*,*i*_(*τ*|*x*), *i*=1,…*m*, are estimated in the *i* th sample. Let *α*=3 in *(12)*, then the weights in *(7)* are
  15 $$ w_{i}(\mathbf{x}_{i},\tau)=\frac{4(1-\tau)^{(5/4)}}{x_{i}},\;x_{i}>1,\;i=1,2,\ldots,n.  $$

The simulation mean squared errors (SMSE) of the estimators *(13)* and *(14)* are:

16 $$\begin{array}{@{}rcl@{}} SMSE(Q_{R}(\tau)) &=&\frac{1}{m}\sum_{i=1}^{m}\int_{1}^{N}(Q_{R,i}(\tau |x)-Q_{y}(\tau |x))^{2}dx; \end{array} $$

17 $$\begin{array}{@{}rcl@{}} SMSE(Q_{W}(\tau)) &=&\frac{1}{m}\sum_{i=1}^{m}\int_{1}^{N}(Q_{W,i}(\tau |x)-Q_{y}(\tau |x))^{2}dx, \end{array} $$

where the true *τ*th conditional quantile *Q*_*y*_(*τ*|*x*) is defined in *(11)*. *N* is a finite *x* value such that the c.d.f. in *(10)* *F*(*N*,*N*)≈1. We take *N*=1000 and the simulation efficiencies (SEFF) are given by

$$SEFF(Q_{W}(\tau))=\frac{SMSE(Q_{R}(\tau))}{SMSE(Q_{W}(\tau))}, $$

where *S* *M* *S* *E*(*Q*_*R*_(*τ*)) and *S* *M* *S* *E*(*Q*_*W*_(*τ*)) are defined in *(16)* and *(17)* respectively.

Table 3 displays the *S* *E* *F* *F*(*Q*_*W*(*f*)_(*τ*)) for varying *τ* values by using the weight in *(15)*. It shows that the *S* *E* *F* *F*(*Q*_*W*(*f*)_(*τ*)) are larger than 1 when *τ*=0.95,…,0.99.

**Table 3:** Simulation mean square errors (SMSE) and efficiencies (SEFF) of estimating *Q*_*y*_(*τ*|*x*),*m*=1000,*n*=300,*N*=1000

*τ*	0.95	0.96	0.97	0.98	0.99
*S* *M* *S* *E*(*Q*_*R*_(*τ*))	2.0533 ×10^8^	2.0103 ×10^8^	2.7731 ×10^8^	5.1199 ×10 ^8^	1.7458 ×10^9^
*S* *M* *S* *E*(*Q*_*W*(*f*)_(*τ*))	1.4939 ×10^8^	1.4146 ×10^8^	2.2462 ×10^8^	4.5520 ×10^8^	1.7161 ×10^9^
*S* *E* *F* *F*(*Q*_*W*(*f*)_(*τ*))	1.6770	1.4211	1.2346	1.1248	1.0174

**Fig. 5:**
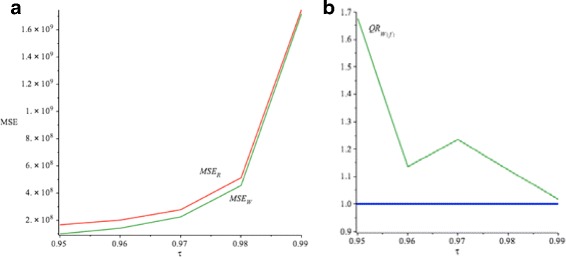
**a** *S* *M* *S* *E*(*Q*_*R*_(*τ*)) is the *red line*, *S* *M* *S* *E*(*Q*_*W*(*f*)_(*τ*)) is the *green line*. **b** *S* *E* *F* *F*(*Q*_*W*(*f*)_(*τ*)) is the *green line*

**Fig. 6:**
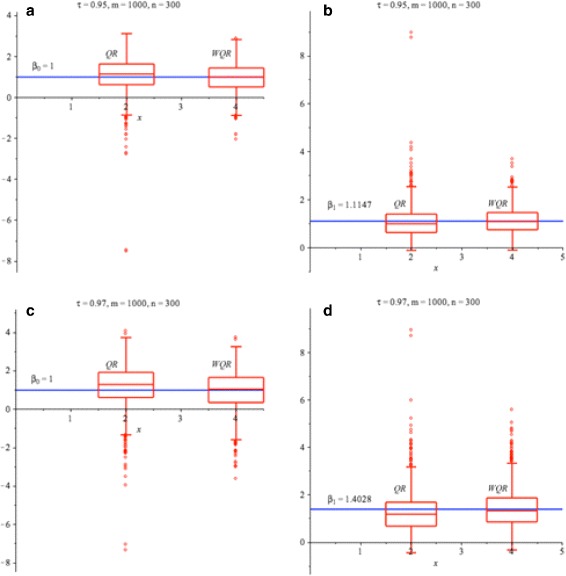
**a** and **c** Box plot comparing the $\widehat {\beta } _{0}$ values of the classical quantile regression *Q*_*R*_(*τ*|*x*) and the weighted quantile regression method *Q*_*W*(*f*)_(*τ*|*x*) to the true *β*_0_=1 (*blue solid line*) for *τ*=0.95 and 0.97 respectively. **b** and **d** Box plot comparing the $\widehat {\beta }_{1}$ values of the classical quantile regression *Q*_*R*_(*τ*|*x*) and the weighted quantile regression *Q*_*W*(*f*)_(*τ*|*x*) to the true *β*_1_=1.1147 and *β*_1_=1.4026 (*blue solid line*) for *τ*=0.95 and 0.97 respectively; *Q*_*R*_(*τ*|*x*) - left box plot and *Q*_*W*(*f*)_(*τ*|*x*) - right box plot

Figure 5 compares the *S* *M* *S* *E*(*Q*_*R*_(*τ*)) with the *S* *M* *S* *E*(*Q*_*W*(*f*)_(*τ*)) for *τ*=0.95,…,0.99. It demonstrates that all *S* *M* *S* *E*(*Q*_*W*(*f*)_(*τ*)) for our proposed weight in *(15)* have smaller values than *S* *M* *S* *E*(*Q*_*R*_(*τ*)). Furthermore, Fig. 6 shows the box plots for estimating the true *β*_0_ and *β*_1_ when *α*=3 by using *Q*_*R*_(*τ*|*x*) and *Q*_*W*(*f*)_(*τ*|*x*) for *τ*=0.95 and 0.97 respectively. It reveals that the proposed *Q*_*W*(*f*)_(*τ*|*x*) is unbiased and produces more accurate $\widehat {\beta }_{W(f)0}(\tau)$ and $\widehat { \beta }_{W(f)1}(\tau)$ estimators to the true *β*_0_ and *β*_1_ for *τ*=0.95 and 0.97. As well, the variances of *Q*_*W*(*f*)_(*τ*|*x*) are smaller relative to *Q*_*R*_(*τ*|*x*) for *τ*=0.95 and 0.97.

**Fig. 7:**
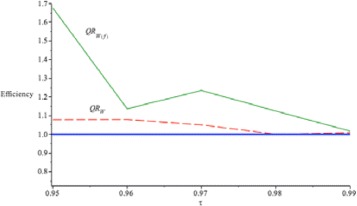
*S* *E* *F* *F*(*Q*_*W*(*f*)_(*τ*)) is the *green* solid line, *S* *E* *F* *F*(*Q*_*W*(1)_(*τ*)) is the red dashed line

Next, we want to compare our simulation results with the following proposed weights presented in Huang et al. (2014) in *(8)*. Table 4 compares the simulation *S* *E* *F* *F*(*Q*_*W*(1)_(*τ*)) by using weight $w_{i}(\mathbf {x}_{i},\tau)=\left \Vert \mathbf {x}_{i}\right \Vert ^{-1}/\sum _{i=1}^{n}\left \Vert \mathbf {x}_{i}\right \Vert ^{-1}$ in *(8)* and *S* *E* *F* *F*(*Q*_*W*(*f*)_(*τ*)) by using weight in *(15)* for different *τ* values. Also, Fig. 7 compares the *S* *E* *F* *F*(*Q*_*W*(1)_(*τ*)) with *S* *E* *F* *F*(*Q*_*W*(*f*)_(*τ*)) with proposed weight in *(15)*. It reveals that the *S* *E* *F* *F*(*Q*_*W*(*f*)_(*τ*)) are larger than 1 and larger than *S* *E* *F* *F*(*Q*_*W*(1)_(*τ*)). Thus, *Q*_*W*(*f*)_(*τ*|*x*) is more efficient than *Q*_*W*(1)_(*τ*|*x*) when *τ*=0.95 and up to 0.99.

**Table 4:** Simulation efficiency (SEFF) of estimating *Q*_*y*_(*τ*|*x*),*m*=1000,*n*=300,*N*=1000

*τ*	0.95	0.96	0.97	0.98	0.99
*S* *E* *F* *F*(*Q*_*W*(1)_(*τ*))	1.0783	1.0729	1.0504	1.0008	1.0086
*S* *E* *F* *F*(*Q*_*W*(*f*)_(*τ*))	1.6770	1.4211	1.2346	1.1248	1.0174

From the overall results of simulation, we can conclude that:

- Table 3, Figs. 5 and 6 show that for *τ*=0.95,…,0.99, the proposed weighted regression *Q*_*W*(*f*)_(*τ*|*x*) with the weight *(15)* is more efficient relative to the regular regression *Q*_*R*_(*τ*|*x*).
- Table 4 and Fig. 7 show that for *τ*=0.95,…,0.99,*Q*_*W*(*f*)_(*τ*|*x*) with the proposed weight *(15)*is more efficient relative to *Q*_*W*(1)_(*τ*|*x*) with $w_{i}(\mathbf {x}_{i},\tau)=\left \Vert \mathbf {x} _{i}\right \Vert ^{-1}/\sum _{i=1}^{n}\left \Vert \mathbf {x}_{i}\right \Vert ^{-1}$ in *(8)*.

## Real examples of applications

In this section, we applied the following three regression models to the Buffalo snowfall and CO_2_ emission examples in Section 1:

- The traditional mean linear regression (LS) estimator $\widehat {\boldsymbol {\beta } } _{LS}$ in *(1)*;
- The regular quantile regression *Q*_*R*_ estimator $\widehat {\boldsymbol \beta }(\tau) $ in *(5)*;
- The proposed weight quantile regression *Q*_*W*_ estimator $\widehat {\boldsymbol \beta }_{W}(\tau)$ in*(6)*with weight *w*_*i*_(**x**_*i*_,*τ*)=*f*_*i*_(*ξ*_*i*_(*τ*,**x**_*i*_)) in *(7)*.

To estimate the proposed local conditional density weight *w*_*i*_(**x**_*i*_,*τ*)=*f*_*i*_(*ξ*_*i*_(*τ*,**x**_*i*_)) in *(7),* we use kernel density estimation (Silverman 1986; Scott 1992).

18 $$ \widehat{w}_{i}\left(\mathbf{x}_{i},\tau \right)=\widehat{f}_{i}\left(\widehat{\xi } _{i}\left(\tau,\mathbf{x}_{i}\right)\right),\text{ where } \widehat{f}\left(y|\mathbf{x} \right)=\frac{\widehat{f}(y,\mathbf{x})}{\widehat{\mu }(\mathbf{x})},  $$

where $\widehat {f}(y,\mathbf {x)}$ is an estimator of the joint density of *y* and **x** **,** and $\widehat {\mu }(\mathbf {x)}$ is an estimator of marginal density of **x**. We estimate the conditional quantile function *ξ*(*τ*|**x**) by inverting an estimated conditional c.d.f. $\widehat {F}(y|\mathbf {x})$ (Li and Racine 2007)

$$\widehat{\xi }(\tau |\mathbf{x})=\widehat{Q}_{y}(\tau |\mathbf{x})=\inf \{y: \widehat{F}(y|\mathbf{x})\geq \tau \}=\widehat{F}^{-1}(\tau |\mathbf{x}), $$

where $\widehat {F}(y|\mathbf {x})$ is the estimated conditional c.d.f. *F*(*y*|**x**).

Note that for a *one*-dimensional random sample *X*_1_,*X*_2_,…,*X*_*n*_ from the distribution *μ*(*x*), a kernel density estimator for *μ*(*x*) is given by

$$\widehat{\mu}(x)=\frac{1}{nh}\sum\limits_{i=1}^{n}K\left(\frac{x-X_{i}}{h}\right), \quad-\infty <x<\infty, $$

where *h* is the window width, and *K*(*x*) is the kernel function, which is a symmetric probability density function with the conditions

$$\int\limits_{-\infty }^{\infty} K(x)dx=1,\int tK(t)dt=0, \int t^{2}K(t)dt=k_{2}\neq 0. $$

The optimal window width can be found by

$$h_{opt}=k_{2}\left\{ \int K(t)^{2}\right\}^{1/5}\left\{ \int \mu^{^{\prime\prime}}(x)^{2}dx\right\}^{-1/5}n^{-1/5}. $$

The *d*-dimensional multivariate kernel density estimator is defined by

$$\widehat{\mu}(\mathbf{x})=\frac{1}{nh^{d}}\sum\limits_{i=1}^{n}K\left\{\frac{\mathbf{x}-\mathbf{X}_{i}}{h}\right\}, $$

where *h* is the window width and the kernel function *K*(**x**) is a function, defined for *d*-dimensional **x** **,** satisfying $ \int \limits _{R^{d}}K(\mathbf {x})d\mathbf {x}=1.$ Fukunaga (1972) suggested

$$\widehat{f}(\mathbf{x})=\frac{(\det \mathbf{S})^{-1/2}}{nh^{d}} \sum\limits_{i=1}^{n}k\left\{ \frac{(\mathbf{x}-\mathbf{X}_{i})^{T}\mathbf{S }^{-1}(\mathbf{x}-\mathbf{X}_{i})}{h^{2}}\right\}, $$

where **S** is the sample covariance matrix of the data, *K* is the normal kernel and the function *k* is given by

$$k(u)=\left(\frac{1}{2\pi}\right)^{d/2}\exp \left(-\frac{u}{2}\right),\quad k(\mathbf{x}^{T}\mathbf{x)}=K(\mathbf{x})=(2\pi)^{-d/2}\exp \left(\frac{1}{2}\mathbf{x}^{T}\mathbf{x}\right). $$

An estimator for the optimal window width *h* will be given by

$$\widehat{h}_{opt}=A(K)n^{-1/(d+4)}, $$

where *A*(*K*)={4/(*d*+2)}^1/(*d*+4)^ is the constant for a multivariate normal kernel.

### 5.1 Buffalo snowfall example

Now, recall the Buffalo snowfall example in Subsection 1.1. We use a polynomial mean regression model *(2)*

$$E\left(y|x,x^{2}\right)=\beta_{0}+\beta_{1}x+\beta_{2}x^{2}, $$

where *y* is the daily snowfall (cm) and *x* is the maximum temperature (°*C*). But the least squares curve only estimates average daily snowfall for a given maximum temperature; it cannot estimate extreme heavy snowfalls. The quantile regression method can estimate high conditional quantile curves and will be shown this Section.

In order to fit the Buffalo snowfall data to the GPD model *(4)*, the data was transformed to $y=\frac {x-\mu }{\sigma },$ where *μ*=5 cm, and then, we used the maximum likelihood estimates (MLEs) of the parameters, $\widehat {\sigma }_{MLE}=5.1552, \widehat {\gamma }_{MLE}=0.2636,$ for the 2-parameter GPD model from the Buffalo snowfall data.

**Fig. 8:**
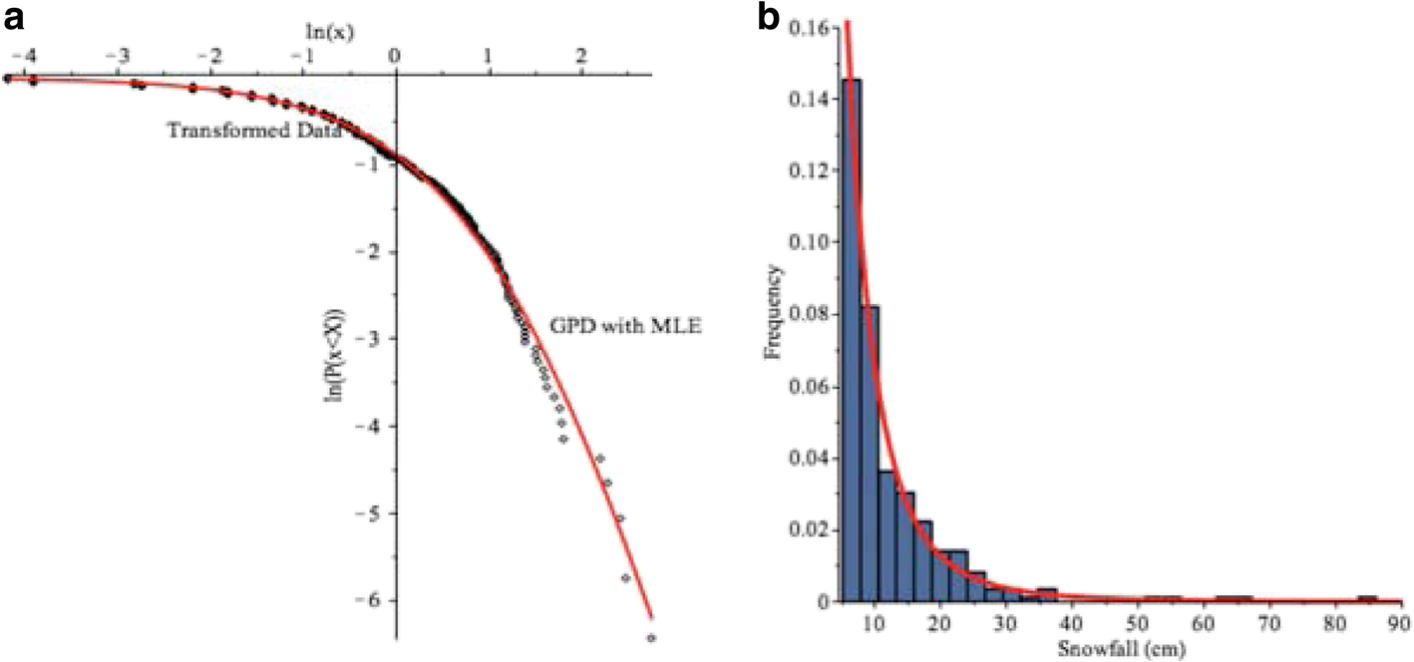
**a** Log-log plot of Buffalo snowfall example. The dots are the data and the *red solid line* is the GPD model. **b** Histogram of the Buffalo snowfall greater than a threshold of 5 cm from January 1994 to January 2015 with the GPD model

Furthermore, Fig. 8(a) and (b) shows the log-log plot and histogram of Buffalo snowfall with GPD model with the MLEs of the parameters. It illustrates that most daily snowfalls in Buffalo are between 0 and 10 centimeters and there are some occurrences of heavy snowfall, such as 50-90 centimeters. The GPD curve follows the shape of the Buffalo snowfall data very well.

Three goodness-of-fit tests are performed: the Kolmogorov-Smirnov test (*K*−*S*) (Kolmogorov 1933), Anderson-Darling test (*A*−*D*) and Cramé r-von-Mises test (*C*−*v*−*M*) (Anderson and Darling 1952) respectively.

$$\begin{array}{@{}rcl@{}} H_{0} &:&F(y)=F^{\ast }(y),\text{ for all values of }y;\\ H_{1} &:&F(y)\neq F^{\ast }(y),\text{ for at least one value of }y, \end{array} $$

where *F*(*y*) is the true but unknown distribution function of the sample and *F* ^∗^(*y*) is the theoretical distribution function, GPD in *(4)*.

In Table 5, the *K*−*S*, *A*−*D* and *C*−*v*−*M* tests show that the GPD model fits the data with a probability of 60.55%, 59.05% and 78.56% respectively.

**Table 5:** The goodness of fit tests for Buffalo snowfall example

*K*−*S*		*A*−*D*		*C*−*v*−*M*	
Test statistic	*p*-value	Test statistic	*p*-value	Test statistic	*p*-value
0.0390	0.6055	0.6631	0.5905	0.0645	0.7856

**Fig. 9:**
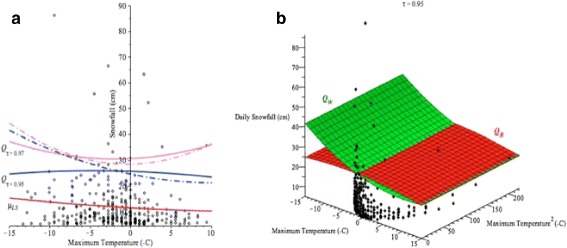
For Buffalo snowfall data, **a** *μ*_*LS*_−*red solid*; Quantile regression at *τ*=0.95 in *blue*, *τ*=0.97 in *pink*, *Q*_*R*_−*solid*, *Q*_*W*_−*dashed*. **b** 3D plot with *Q*_*R*_−*red* and *Q*_*W*_−*green* at *τ*=0.95

Instead of using model *(2),* we use the following quantile regression model:

$$Q_{y}(\tau |x)=\beta_{0}(\tau)+\beta_{1}x(\tau)+\beta_{2}(\tau)x^{2}, $$

where we use the estimated weight $\widehat {w}_{i}(\mathbf {x}_{i},\tau)= \widehat {f}_{i}(\widehat {\xi }(\tau))$ in *(18)*. Figure 9 shows the scatter plot of the daily snowfall with the fitted *μ*_*LS*_,*Q*_*R*_ and *Q*_*W*_ curves at two high 0.95th and 0.97th quantiles. It is interesting to note that at the 0.95th and 0.97th quantiles, the *Q*_*R*_ and *Q*_*W*_ curves appear to fit the data. Table 6 lists the estimated Buffalo snowfall quantile values at a given maximum temperature for *τ*=0.95 and 0.97. Both Fig. 9 and Table 6 demonstrate that when quantiles are high, *Q*_*W*_ have heavier snowfall than *Q*_*R*_.

**Table 6:** Buffalo daily snowfalls (cm) at high quantile using *Q*_*R*_ and *Q*_*W*_

	*τ*=0.95		*τ*=0.97	
Temperature (°*C*)	*Q*_*R*_	*Q*_*W*_	*Q*_*R*_	*Q*_*W*_
–15	24.72	41.57	37.38	44.38
–10	25.65	34.37	33.19	35.42
–5	26.00	28.74	30.98	30.15
0	25.76	24.69	30.73	28.58
5	24.93	22.21	32.47	30.70
10	23.52	21.30	36.17	36.52

**Fig. 10:**
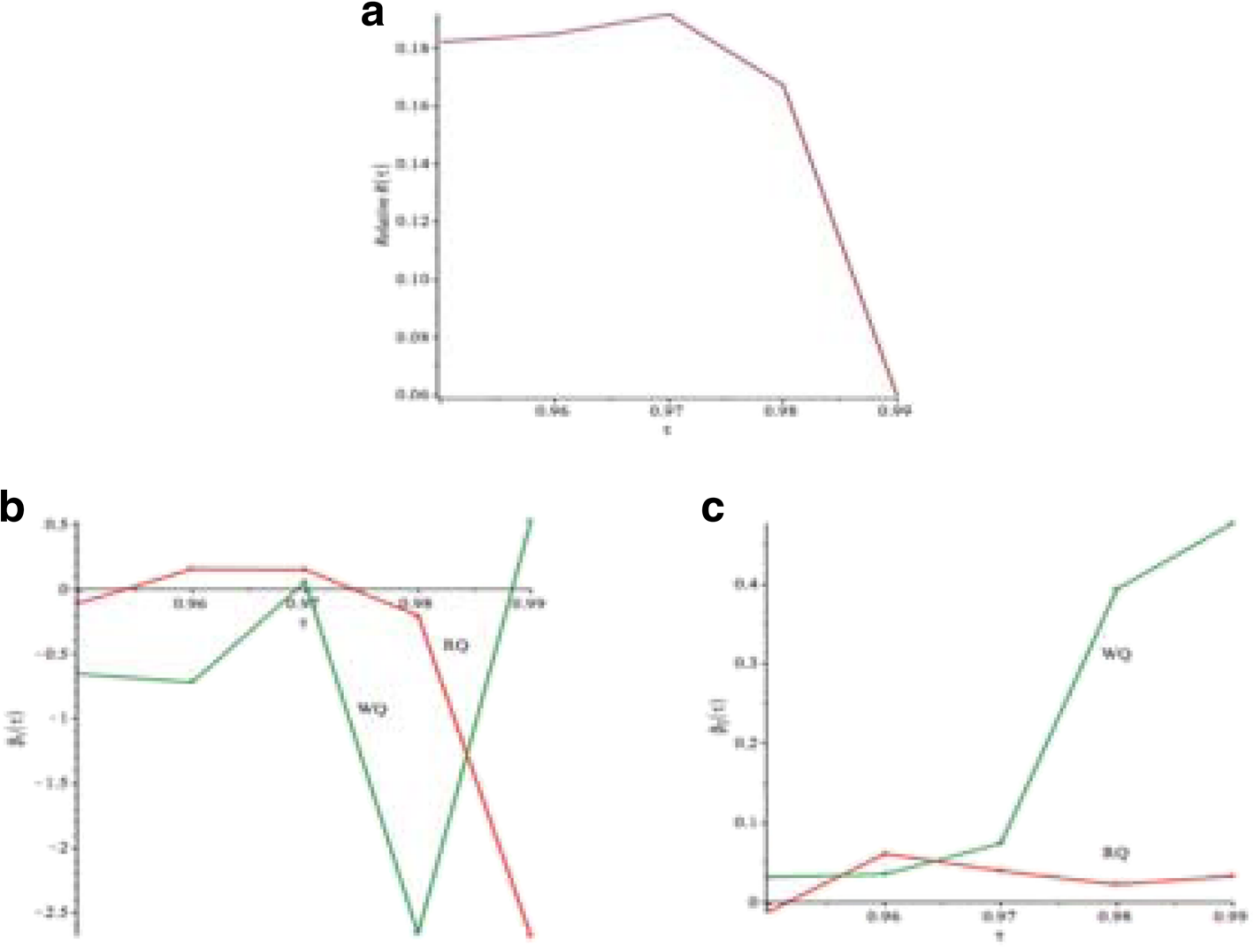
Buffalo snowfall example: **a** Relative *R*(*τ*).**b** Comparison of $\widehat {\beta }_{1}(\tau).$ **c** Comparison of $\widehat { \beta }(\tau)$. *Q*_*R*_−*red* *solid line*, *Q*_*W*_−*green solid line*

Figure 10(a) and Table 7 show the values of the Relative *R*(*τ*) in *(9)* for given *τ*=0.95,…,0.99. We note that *R*(*τ*)>0, which means that *V*_*weighted*_(*τ*)<*V*_*regular*_(*τ*), and the *Q*_*W*_ is a better fit to the data than the *Q*_*R*_. Figure 10(b), (c) and Table 8 show the values of $\widehat {\beta }_{1}(\tau)$ and $\widehat {\beta }_{2}(\tau)$. The values of $\widehat {\beta }_{1}(\tau)$ and $\widehat {\beta }_{2}(\tau)$ are consistent with Fig. 9(a), (b) and Table 6.

**Table 7:** Relative *R*(*τ*) values for the Buffalo snowfall example

	*τ*=0.95	*τ*=0.96	*τ*=0.97	*τ*=0.98	*τ*=0.99
Relative *R*(*τ*)	0.1824	0.1851	0.1919	0.1673	0.0595

**Table 8:** Coefficients of the *Q*_*R*_,*Q*_*W*_ and LS *μ*_*LS*_ regression for buffalo snowfall example

*τ*	Weight	$\widehat {\beta }_{0}{ (\tau)}$	$\widehat {\beta }_{1}{ (\tau)}$	$\widehat {\beta }_{2}{(\tau)}$
LS	−	11.5280	–0.1777	0.0053
0.95	*Q*_*R*_	25.7589	–10.1068	–0.0117
	*Q*_*W*_	24.6887	–0.6538	0.0315
0.96	*Q*_*R*_	28.7869	0.1543	0.0610
	*Q*_*W*_	24.8614	–0.7200	0.0352
0.97	*Q*_*R*_	30.7341	0.1488	0.0395
	*Q*_*W*_	28.5776	0.0551	0.0739
0.98	*Q*_*R*_	35.5582	–0.2039	0.0223
	*Q*_*W*_	25.8718	–2.6593	0.3937
0.99	*Q*_*R*_	57.8614	–2.6793	0.0330
	*Q*_*R*_	48.5464	0.5261	0.4768

The proposed weighted quantile regression *Q*_*W*_ predicts that for moderate temperatures, such as 5 °*C* to 10 °*C*, it is likely to have small snowfalls in Buffalo, and for every low temperatures, such as −15 °*C* to 0 °*C*, it is more likely to have heavy snowfalls that may cause damage. Predicting heavy snowfalls is related to cold weather forecasts. Quantile regression is useful for predicting extreme heavy snowfalls.

### 5.2 CO_2_ emission example

In Subsection 1.2, there is a relationship between ln(GDP) *x*_1_ and ln(E.C.) *x*_2_ and CO_2_ emissions per capita *y*. The least squares estimate is:

$$\mu_{LS}=-22.5009+2.0708x_{1}+1.2998x_{2}. $$

The quantile regression method can estimate high conditional quantile curves and will be shown in detail in this Section.

**Fig. 11:**
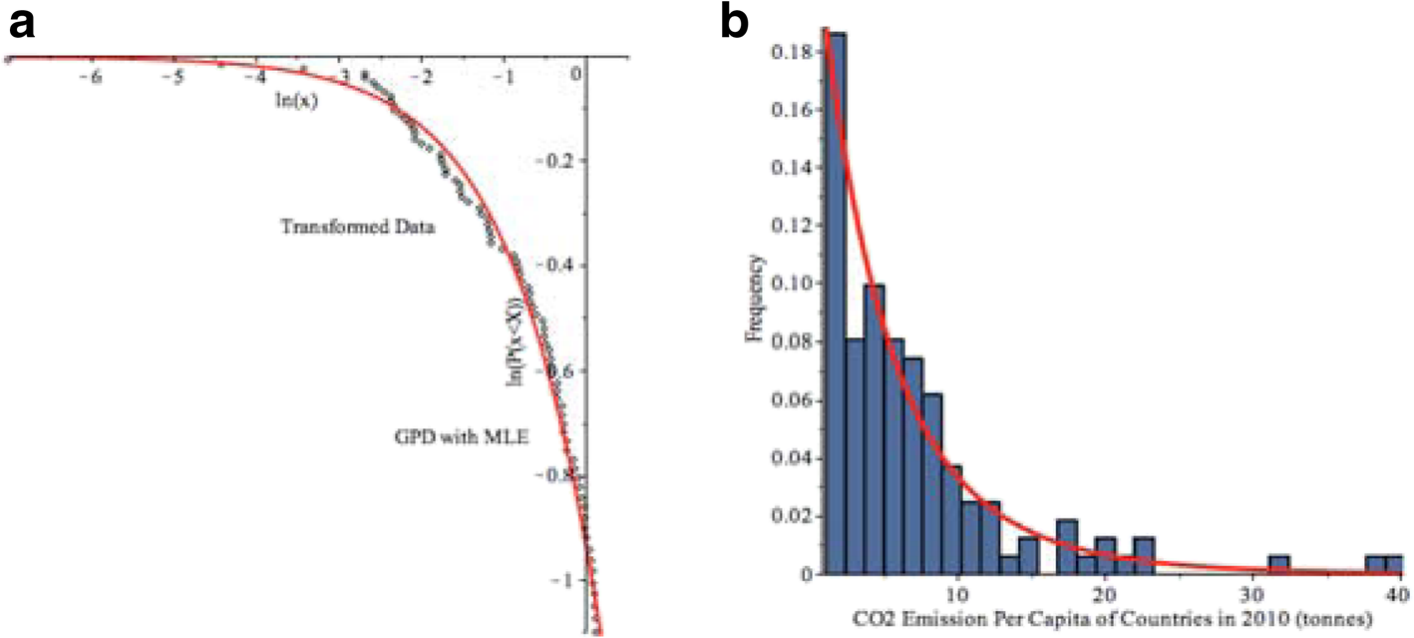
**a** Log-log plot of CO_2_ emission example. The dots are the data and the *red solid line* is the GPD model. **b** Histogram of CO _2_ emission per capita of 123 countries in 2010 (tonnes) greater than the threshold of 1 tonne with GPD model in *(4)*

Similar to the Buffalo snowfall example, we fit the GPD model in *(4)* with MLEs of the parameters, $\widehat {\sigma }_{MLE}=5.3011$, $\widehat {\gamma }_{MLE}=0.1234,$ to the CO_2_ emission data, which is demonstrated in Fig. 11(a), (b) by the log-log plot and histogram. The GPD model follows the shape of the CO_2_ emission data very well. Table 9 shows the results of the three goodness-of-fit tests.

**Table 9:** The goodness of fit tests for CO_2_ emission example

*K*−*S*		*A*−*D*		*C*−*v*−*M*	
Test statistic	*p*-value	Test statistic	*p*-value	Test statistic	*p*-value
0.0443	0.8397	0.3619	0.8855	0.0517	0.8662

We use the proposed weight $\widehat {w}_{i}(x_{i},\tau)=\widehat {f}_{i}(\widehat {\xi }(\tau))$ in *(18)* on the quantile regression model:

$$Q_{y}\left(\tau |x_{1},x_{2}\right)=\beta_{0}(\tau)+\beta_{1}(\tau)x_{1}+\beta_{2}(\tau)x_{2}. $$

**Fig. 12:**
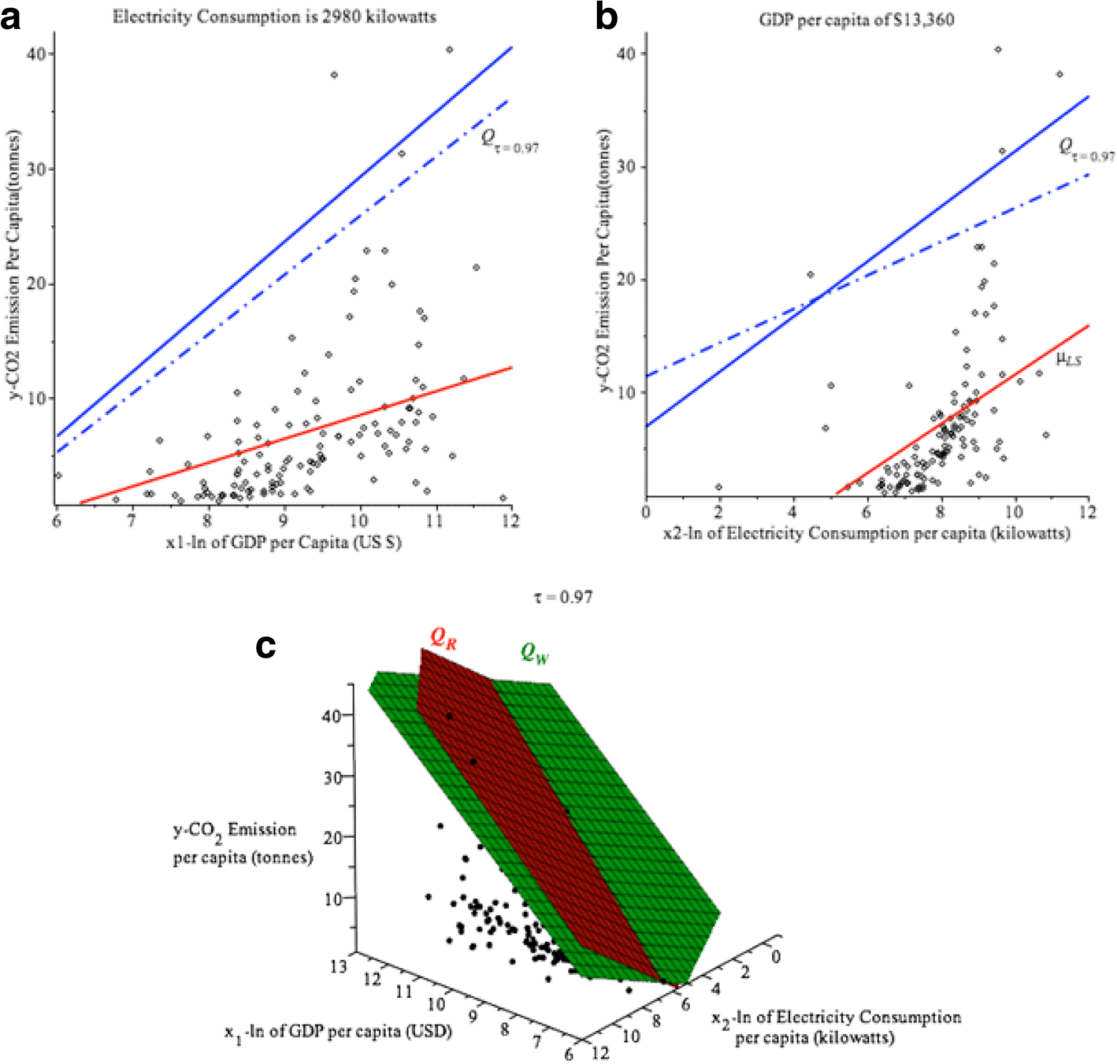
**a** Quantile regression of the CO_2_ emission vs ln(GDP) when the country’s E.C. is 2980.96 kilowatts at *τ*=0.97.*Q*_*R*_−*solid blue*, *Q*_*W*_−dashed blue, *μ*_*LS*_−*red solid line*. **b** Quantile regression of the CO_2_ emission vs ln(E.C.) when the country’s GDP is $13,359.73 at *τ*=0.97. *Q*_*R*_−*solid blue*, *Q*_*W*_−*dashed blue*, *μ*_*LS*_−*red solid line*. **c** 3D scatter plot with *Q*_*R*_−*red* and *Q*_*W*_−*green* for *τ*=0.97

Figure 12(a) shows the scatter plot of CO_2_ emission vs ln(GDP) when the country’s E.C. is 2980.96 kilowatts with the fitted *μ*_*LS*_,*Q*_*R*_ and *Q*_*W*_ curves at the 0.97th quantile. Figure 12(b) shows the scatter plot of CO_2_ emission vs ln(E.C.) when the country’s GDP is $13,359.73 with the fitted *μ*_*LS*_,*Q*_*R*_ and *Q*_*W*_ curves at the 0.97th quantile. Figure 12(c) shows the 3D scatter plot with *Q*_*R*_ (red) and *Q*_*W*_ (green) of CO_2_ emission given the ln(GDP) and ln(E.C.) at *τ*=0.97. It is important to note that the *μ*_*LS*_ is the red solid line and the *Q*_*R*_ and *Q*_*W*_ quantile regression lines appear to fit the data. In general, the *Q*_*W*_ line produces a different estimated CO_2_ emissions than *Q*_*R*_ curve at high quantiles. Tables 10 and 11 provide details about countries’ CO_2_ emission at high quantile (*τ*=0.97) when the countries consume 2980.96 kilowatts of electricity and have a GDP of $13,359.73 respectively.

**Table 10:** CO_2_ emission per capita high quantile given ln(GDP) estimators *Q*_*R*_ and *Q*_*W*_ at 2980.96 Kilowatts of electricity consumed per capita

	*τ*=0.97	
ln of GDP per capita ($)	*Q*_*R*_	*Q*_*W*_
7.5	15.2181	13.0840
8	18.0437	15.6591
8.5	20.8693	18.2342
9	23.6950	20.9093
9.5	26.5206	23.3844
10	29.3462	25.9595
10.5	32.1718	28.5346
11	34.9975	31.1097
11.5	37.8231	33.5848
12	40.6487	36.2599

**Table 11:** CO_2_ Emission per capita high quantile given ln(E.C.) estimators Q _*R*_ and Q _*W*_ at GDP per capita of $13,359.73

ln of Electricity Consumption per capita (kilowatts)	*τ*=0.97	
	*Q*_*R*_	*Q*_*W*_
0	6.9775	11.4376
2	11.8632	14.4243
4	16.7490	17.4110
6	21.6348	20.3977
8	26.5206	23.3844
10	31.4064	26.3711
12	36.2921	29.3578

**Fig. 13:**
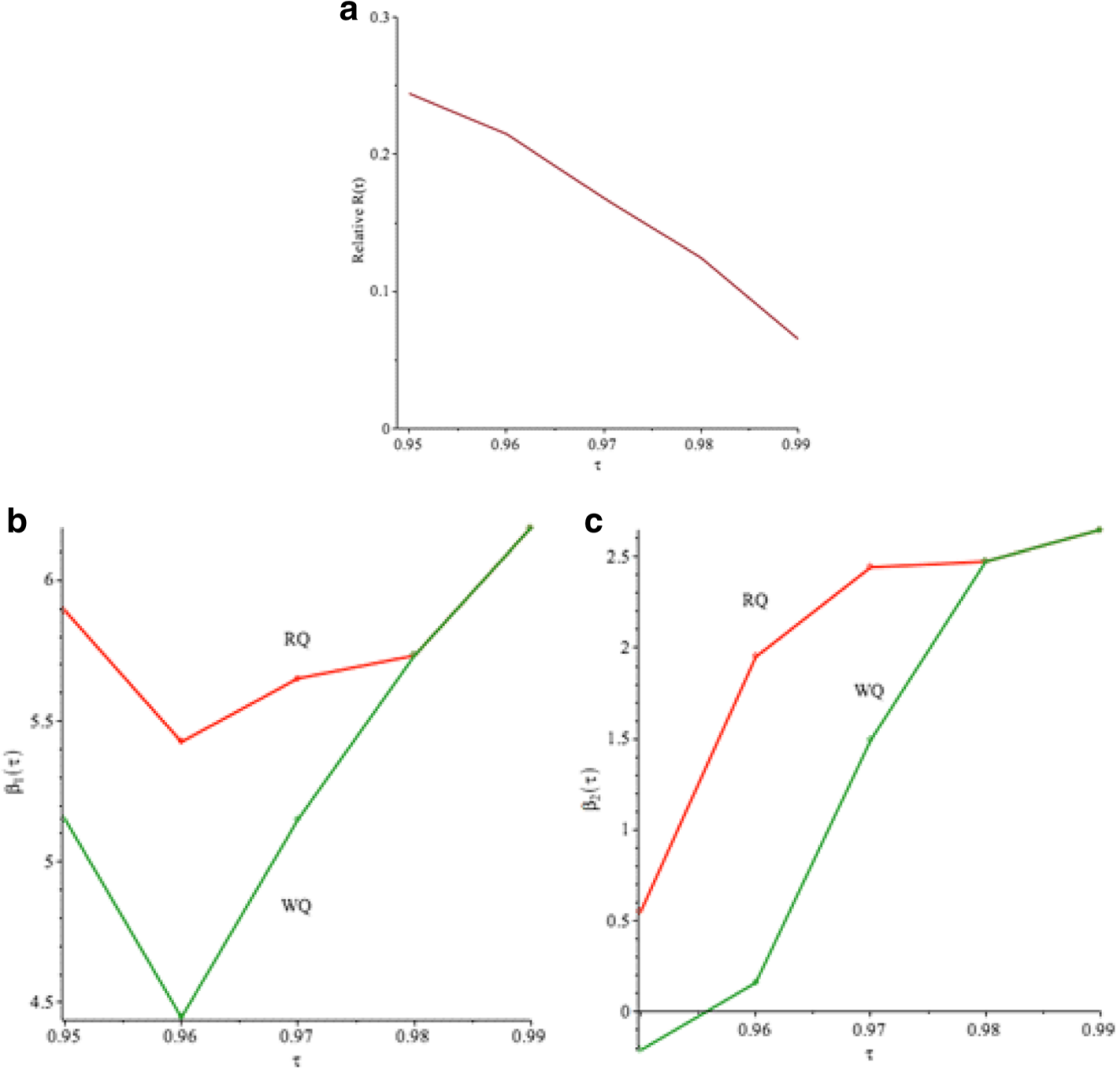
**a** Relative *R*(*τ*).**b** Comparison of $\widehat {\beta }_{1}(\tau).$ **c** Comparison of $\widehat {\beta }_{2}(\tau)$. *Q*_*R*_−*red line*, *Q*_*W*_−*green line* for CO_2_ emission example

Figure 13(a) shows the Relative *R*(*τ*), which is defined in *(9)* and Table 12 shows the values for Relative *R*(*τ*) for *τ*≥0.95. All values of Relative *R*(*τ*) are larger than 0, which signifies that *V*_*weighted*_(*τ*)<*V*_*regular*_(*τ*) and as well, it suggests that the weighted quantile regression model *Q*_*W*_ is a better fit to the CO_2_ emission data than the regular quantile regression model *Q*_*R*_. Figure 13(b) and (c) shows the values of $\widehat {\beta }_{1}(\tau)$ and $\widehat {\beta }_{2}(\tau)$ for a given *τ* respectively, which is also shown in Table 13. The values of $\widehat {\beta }_{1}(\tau)$ and $\widehat {\beta }_{2}(\tau)$ are consistent with Tables 10 and 11.

**Table 12:** Relative *R*(*τ*) values for CO_2_ emission example

	*τ*=0.95	*τ*=0.96	*τ*=0.97	*τ*=0.98	*τ*=0.99
Relative *R*(*τ*)	0.24425	0.21478	0.16810	0.12459	0.06521

**Table 13:** Coefficients of the *Q*_*R*_,*Q*_*W*_ and LS *μ*_*LS*_ regression for CO_2_ emission example

*τ*	Weight	$\widehat {\beta }_{0}{ (\tau)}$	$\widehat {\beta }_{1}{ (\tau)}$	$\widehat {\beta }_{2}{(\tau)}$
LS	−	–22.5009	2.0708	1.2998
0.95	*Q*_*R*_	–41.6856	5.8924	0.5527
	*Q*_*W*_	–29.9131	5.1521	–0.2094
0.96	*Q*_*R*_	–44.8147	5.4258	1.9505
	*Q*_*W*_	–24.5504	4.4470	0.1609
0.97	*Q*_*R*_	–46.7095	5.6513	2.4429
	*Q*_*W*_	–37.4893	5.1502	1.4934
0.98	*Q*_*R*_	–47.4004	5.7323	2.4739
	*Q*_*W*_	–47.4004	5.7323	2.4739
0.99	*Q*_*R*_	–51.2657	6.1856	2.6475
	*Q*_*W*_	–51.2657	6.1856	2.6475

It can be concluded that countries with higher gross domestic product and higher amounts of electricity produce higher CO_2_ emissions. Since CO _2_ is not destroyed over time, it can remain in the atmosphere for thousands of years due to the very slow process by which carbon is transferred to ocean sediments. As a result, countries should monitor their CO_2_ emissions in order to prevent further damages to the environment. Countries can consider producing more energy from renewable sources, such as wind, solar, hydro and geothermal heat and using fuels with lower carbon content to reduce carbon emissions.

## Conclusions

After the studies above, we can conclude:

- Traditional mean regression are concerned with estimating the conditional mean by using the L_2_-loss function. Quantile regression with a L_1_- loss function overcomes the limitations of traditional mean regression. It gives estimates of *τ*th conditional quantiles besides the measures of central tendency. Estimation of high conditional quantiles is very useful for the analysis of extreme events.
- The proposed weighted quantile regression method with the local conditional density as the weight has good mathematical asymptotic properties.
- The Monte Carlo computational simulation results show that the proposed weighted quantile regression with the local conditional density as the weight is more efficient relative to the classical quantile regression and some existing weighted quantile regression estimators.
- The proposed weighted quantile regression can be used to predict extreme values of snowfall and CO_2_ emission real world examples. In the Buffalo snowfall example, communities can use the information that quantile regression provides to prevent car accidents on roads, overexertion, and collapsing of homes. In the CO_2_ emission example, the countries’ increase in gross domestic product and electricity consumption will likely cause an increase in the CO_2_ emissions. CO_2_ emission levels should be monitored to reduce the amount of carbon dioxide in the atmosphere and its long term effects.
- It is difficult to estimate the proposed conditional density weight. The nonparametric kernel density estimation method is successful in this paper. Further studies on optimal weights are suggested.
